# [(*Z*)-*O*-Isopropyl *N*-phenyl­thio­car­bam­ato-κ*S*](tricyclo­hexyl­phosphine-κ*P*)gold(I)

**DOI:** 10.1107/S1600536810009906

**Published:** 2010-03-20

**Authors:** Primjira P. Tadbuppa, Edward R. T. Tiekink

**Affiliations:** a Department of Chemistry, National University of Singapore, Singapore 117543; b Department of Chemistry, University of Malaya, 50603 Kuala Lumpur, Malaysia

## Abstract

The Au atom in the title compound, [Au(C_10_H_12_NOS)(C_18_H_33_P)], is coordinated within an *S*,*P*-donor set that defines a slightly distorted linear geometry [S—Au—P = 174.54 (2)°], with the distortion due in part to a close intra­molecular Au⋯O contact [3.1702 (16) Å]. In the crystal structure, mol­ecules are arranged into supra­molecular chains along the *b* axis mediated by C—H⋯O inter­actions.

## Related literature

For the structural systematics and luminescence properties of phosphinegold(I) carbonimidothio­ates, see: Ho *et al.* (2006 ▶); Ho & Tiekink (2007 ▶); Kuan *et al.* (2008 ▶). For the synthesis, see Hall *et al.* (1993 ▶).
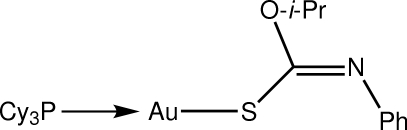


## Experimental

### 

#### Crystal data

[Au(C_10_H_12_NOS)(C_18_H_33_P)]
*M* *_r_* = 671.65
Orthorhombic, 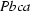

*a* = 19.3312 (10) Å
*b* = 15.1116 (7) Å
*c* = 19.811 (1) Å
*V* = 5787.3 (5) Å^3^
*Z* = 8
Mo *K*α radiation
μ = 5.23 mm^−1^
*T* = 193 K
0.23 × 0.18 × 0.16 mm

#### Data collection

Bruker SMART CCD diffractometer
Absorption correction: multi-scan (*SADABS*; Bruker, 2000 ▶) *T*_min_ = 0.544, *T*_max_ = 1
39425 measured reflections
6645 independent reflections
5481 reflections with *I* > 2σ(*I*)
*R*_int_ = 0.040

#### Refinement

*R*[*F* ^2^ > 2σ(*F* ^2^)] = 0.021
*wR*(*F* ^2^) = 0.050
*S* = 1.03
6645 reflections
300 parameters
H-atom parameters constrained
Δρ_max_ = 0.99 e Å^−3^
Δρ_min_ = −0.69 e Å^−3^

### 

Data collection: *SMART* (Bruker, 2000 ▶); cell refinement: *SAINT* (Bruker, 2000 ▶); data reduction: *SAINT*; program(s) used to solve structure: *PATTY* in *DIRDIF92* (Beurskens *et al.*, 1992 ▶); program(s) used to refine structure: *SHELXL97* (Sheldrick, 2008 ▶); molecular graphics: *ORTEP-3* (Farrugia, 1997 ▶) and *DIAMOND* (Brandenburg, 2006 ▶); software used to prepare material for publication: *publCIF* (Westrip, 2010 ▶).

## Supplementary Material

Crystal structure: contains datablocks global, I. DOI: 10.1107/S1600536810009906/ez2204sup1.cif

Structure factors: contains datablocks I. DOI: 10.1107/S1600536810009906/ez2204Isup2.hkl

Additional supplementary materials:  crystallographic information; 3D view; checkCIF report

## Figures and Tables

**Table 1:** Selected bond lengths (Å)

Au—P1	2.2641 (6)
Au—S1	2.2998 (6)

**Table 2:** Hydrogen-bond geometry (Å, °)

*D*—H⋯*A*	*D*—H	H⋯*A*	*D*⋯*A*	*D*—H⋯*A*
C4—H4⋯O1^i^	0.95	2.48	3.367 (3)	156

Symmetry code: (i) 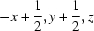.

**Fig. 1.**
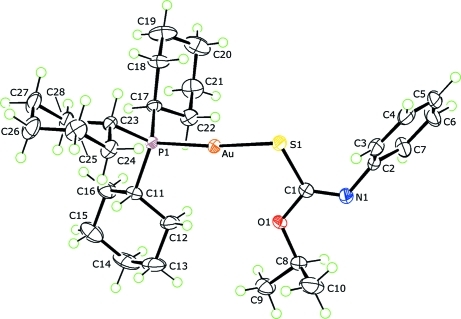
Molecular structure of (I) showing atom-labelling scheme and displacement ellipsoids at the 35% probability level.

**Fig. 2.**
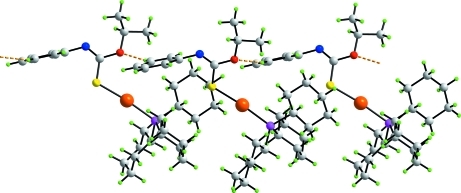
A view of a supramolecular chain in (I) aligned along the b axis with the C–H···O interactions shown as orange dashed lines. Colour code: Au, orange; S, yellow; P, pink; O, red; N, blue; C, grey; and H, green.

## supplementary crystallographic information

### Comment

Systematic structural studies of molecules with the general formula R_3_PAu[SC(OR')═NR''], for R, R' and R'' = alkyl and aryl, are motivated by crystal engineering and luminescence considerations (Ho *et al.* 2006; Ho & Tiekink, 2007; Kuan *et al.*, 2008), and led to the synthesis and characterisation of the title compound, (I).

The gold atom in (I) exists within an *SP* donor set defined by the phosphine-*P* and thiolate-*S* atoms, Table 1 and Fig. 1. The carbonimidothioate ligand is functioning as a thiolate as seen by the magnitudes of the C1—S1 [1.755 (2) Å] and C1═N1 [1.263 (3) Å] bond distances. The coordination geometry is distorted from the ideal linear [S—Au—P = 174.54 (2) °] owing to the close approach of the O1 atom [3.1702 (16) Å].

The most prominent interactions in the crystal structure are of the type C–H···O, Table 2, and these lead to the formation of supramolecular chains with glide symmetry along the *b* axis, Fig. 2.

### Experimental

Compound (I) was prepared following the standard literature procedure from the reaction of Cy_3_PAuCl and (*i*-Pr)OC(═S)N(H)Ph in the presence of NaOH (Hall *et al.*, 1993). Crystals were obtained by the slow evaporation of a CH_2_Cl_2_/hexane (3/1) solution held at room temperature.

### Refinement

The H atoms were geometrically placed (C—H = 0.95-1.00 Å) and refined as riding with *U*_iso_(H) = 1.2-1.5*U*_eq_(C).

### Crystal data

**Table:**

[Au(C_10_H_12_NOS)(C_18_H_33_P)]	*F*(000) = 2704
*M**_r_* = 671.65	*D*_x_ = 1.542 Mg m^−^^3^
Orthorhombic, *P**b**c**a*	Mo *K*α radiation, λ = 0.71069 Å
Hall symbol: -P 2ac 2ab	Cell parameters from 6370 reflections
*a* = 19.3312 (10) Å	θ = 2.3–30.0°
*b* = 15.1116 (7) Å	µ = 5.23 mm^−^^1^
*c* = 19.811 (1) Å	*T* = 193 K
*V* = 5787.3 (5) Å^3^	Block, colourless
*Z* = 8	0.23 × 0.18 × 0.16 mm

### Data collection

**Table:**

Bruker SMART CCD diffractometer	6645 independent reflections
Radiation source: fine-focus sealed tube	5481 reflections with *I* > 2σ(*I*)
graphite	*R*_int_ = 0.040
ω scans	θ_max_ = 27.5°, θ_min_ = 2.0°
Absorption correction: multi-scan (*SADABS*; Bruker, 2000)	*h* = −25→23
*T*_min_ = 0.544, *T*_max_ = 1	*k* = −19→12
39425 measured reflections	*l* = −25→25

### Refinement

**Table:**

Refinement on *F*^2^	Primary atom site location: structure-invariant direct methods
Least-squares matrix: full	Secondary atom site location: difference Fourier map
*R*[*F*^2^ > 2σ(*F*^2^)] = 0.021	Hydrogen site location: inferred from neighbouring sites
*wR*(*F*^2^) = 0.050	H-atom parameters constrained
*S* = 1.03	*w* = 1/[σ^2^(*F*_o_^2^) + (0.0229*P*)^2^] where *P* = (*F*_o_^2^ + 2*F*_c_^2^)/3
6645 reflections	(Δ/σ)_max_ = 0.005
300 parameters	Δρ_max_ = 0.99 e Å^−^^3^
0 restraints	Δρ_min_ = −0.69 e Å^−^^3^

### Special details

**Table:**

Geometry. All s.u.'s (except the s.u. in the dihedral angle between two l.s. planes) are estimated using the full covariance matrix. The cell s.u.'s are taken into account individually in the estimation of s.u.'s in distances, angles and torsion angles; correlations between s.u.'s in cell parameters are only used when they are defined by crystal symmetry. An approximate (isotropic) treatment of cell s.u.'s is used for estimating s.u.'s involving l.s. planes.
Refinement. Refinement of *F*^2^ against ALL reflections. The weighted *R*-factor wR and goodness of fit *S* are based on *F*^2^, conventional *R*-factors *R* are based on *F*, with *F* set to zero for negative *F*^2^. The threshold expression of *F*^2^ > 2σ(*F*^2^) is used only for calculating *R*-factors(gt) etc. and is not relevant to the choice of reflections for refinement. *R*-factors based on *F*^2^ are statistically about twice as large as those based on *F*, and *R*- factors based on ALL data will be even larger.

### Fractional atomic coordinates and isotropic or equivalent isotropic displacement parameters (Å^2^)

**Table:**

	*x*	*y*	*z*	*U*_iso_*/*U*_eq_	
Au	0.187999 (5)	0.453847 (6)	0.925784 (4)	0.01954 (4)	
S1	0.20150 (3)	0.58446 (4)	0.86767 (3)	0.02664 (14)	
P1	0.17856 (3)	0.33212 (4)	0.99177 (3)	0.01871 (13)	
O1	0.14511 (8)	0.48712 (10)	0.77343 (8)	0.0226 (4)	
N1	0.13197 (11)	0.63489 (12)	0.75576 (10)	0.0248 (5)	
C1	0.15504 (12)	0.57284 (15)	0.79206 (12)	0.0210 (5)	
C2	0.14430 (13)	0.72399 (14)	0.77403 (12)	0.0233 (5)	
C3	0.20992 (13)	0.76015 (16)	0.77533 (13)	0.0280 (6)	
H3	0.2489	0.7237	0.7662	0.034*	
C4	0.21951 (15)	0.84864 (16)	0.78978 (13)	0.0318 (6)	
H4	0.2650	0.8726	0.7905	0.038*	
C5	0.16365 (16)	0.90265 (17)	0.80318 (15)	0.0376 (7)	
H5	0.1703	0.9633	0.8140	0.045*	
C6	0.09806 (16)	0.86735 (16)	0.80066 (16)	0.0403 (7)	
H6	0.0592	0.9041	0.8093	0.048*	
C7	0.08822 (14)	0.77876 (16)	0.78567 (15)	0.0331 (6)	
H7	0.0427	0.7554	0.7834	0.040*	
C8	0.11414 (14)	0.47124 (16)	0.70740 (13)	0.0285 (6)	
H8	0.0726	0.5099	0.7016	0.034*	
C9	0.09212 (15)	0.37553 (16)	0.70929 (15)	0.0360 (7)	
H9A	0.1330	0.3378	0.7146	0.054*	
H9B	0.0684	0.3605	0.6671	0.054*	
H9C	0.0606	0.3661	0.7474	0.054*	
C10	0.16562 (18)	0.4903 (2)	0.65267 (16)	0.0490 (8)	
H10A	0.1814	0.5517	0.6564	0.073*	
H10B	0.1439	0.4812	0.6085	0.073*	
H10C	0.2053	0.4503	0.6573	0.073*	
C11	0.14793 (13)	0.23346 (15)	0.94552 (13)	0.0233 (5)	
H11	0.1904	0.2027	0.9289	0.028*	
C12	0.10620 (14)	0.25767 (17)	0.88274 (14)	0.0334 (6)	
H12A	0.0627	0.2872	0.8964	0.040*	
H12B	0.1330	0.2997	0.8547	0.040*	
C13	0.08930 (16)	0.1754 (2)	0.84138 (16)	0.0463 (8)	
H13A	0.0602	0.1924	0.8024	0.056*	
H13B	0.1327	0.1495	0.8237	0.056*	
C14	0.05156 (16)	0.1066 (2)	0.88319 (18)	0.0551 (10)	
H14A	0.0057	0.1299	0.8965	0.066*	
H14B	0.0441	0.0528	0.8557	0.066*	
C15	0.09231 (18)	0.0828 (2)	0.94590 (18)	0.0515 (9)	
H15A	0.1361	0.0537	0.9326	0.062*	
H15B	0.0654	0.0403	0.9734	0.062*	
C16	0.10861 (15)	0.16520 (16)	0.98846 (15)	0.0357 (7)	
H16A	0.1371	0.1482	1.0279	0.043*	
H16B	0.0650	0.1916	1.0053	0.043*	
C17	0.11942 (13)	0.35252 (15)	1.06315 (11)	0.0214 (5)	
H17	0.1117	0.2954	1.0874	0.026*	
C18	0.15046 (15)	0.41908 (19)	1.11282 (14)	0.0356 (7)	
H18A	0.1620	0.4746	1.0888	0.043*	
H18B	0.1937	0.3947	1.1322	0.043*	
C19	0.09894 (18)	0.4387 (2)	1.16965 (17)	0.0554 (9)	
H19A	0.0914	0.3841	1.1963	0.066*	
H19B	0.1190	0.4838	1.2001	0.066*	
C20	0.03053 (18)	0.4714 (2)	1.14351 (18)	0.0548 (9)	
H20A	0.0371	0.5290	1.1206	0.066*	
H20B	−0.0016	0.4805	1.1818	0.066*	
C21	−0.00063 (15)	0.4056 (2)	1.09443 (15)	0.0417 (7)	
H21A	−0.0441	0.4300	1.0756	0.050*	
H21B	−0.0119	0.3501	1.1185	0.050*	
C22	0.04988 (13)	0.38596 (17)	1.03702 (13)	0.0314 (6)	
H22A	0.0294	0.3408	1.0068	0.038*	
H22B	0.0573	0.4405	1.0103	0.038*	
C23	0.26327 (12)	0.30306 (15)	1.02821 (12)	0.0230 (5)	
H23	0.2764	0.3532	1.0585	0.028*	
C24	0.31875 (12)	0.29918 (18)	0.97299 (14)	0.0284 (6)	
H24A	0.3078	0.2507	0.9411	0.034*	
H24B	0.3188	0.3555	0.9474	0.034*	
C25	0.39004 (14)	0.28369 (19)	1.00344 (15)	0.0395 (7)	
H25A	0.4033	0.3355	1.0311	0.047*	
H25B	0.4245	0.2773	0.9668	0.047*	
C26	0.39063 (15)	0.2002 (2)	1.04759 (16)	0.0442 (8)	
H26A	0.3820	0.1476	1.0190	0.053*	
H26B	0.4368	0.1934	1.0686	0.053*	
C27	0.33607 (15)	0.2054 (2)	1.10215 (15)	0.0396 (7)	
H27A	0.3472	0.2546	1.1333	0.047*	
H27B	0.3360	0.1497	1.1285	0.047*	
C28	0.26457 (14)	0.22035 (17)	1.07165 (13)	0.0310 (6)	
H28A	0.2516	0.1685	1.0438	0.037*	
H28B	0.2301	0.2262	1.1083	0.037*	

### Atomic displacement parameters (Å^2^)

**Table:**

	*U*^11^	*U*^22^	*U*^33^	*U*^12^	*U*^13^	*U*^23^
Au	0.02220 (6)	0.01907 (6)	0.01734 (6)	0.00080 (4)	−0.00015 (4)	0.00148 (3)
S1	0.0369 (4)	0.0185 (3)	0.0245 (3)	−0.0044 (3)	−0.0083 (3)	0.0021 (2)
P1	0.0202 (3)	0.0198 (3)	0.0161 (3)	−0.0002 (2)	−0.0012 (2)	0.0001 (2)
O1	0.0274 (9)	0.0162 (8)	0.0242 (9)	−0.0010 (7)	−0.0049 (8)	−0.0033 (7)
N1	0.0287 (12)	0.0197 (10)	0.0259 (12)	−0.0001 (9)	−0.0057 (9)	0.0009 (8)
C1	0.0187 (12)	0.0212 (12)	0.0232 (14)	−0.0003 (10)	0.0014 (10)	−0.0016 (10)
C2	0.0310 (14)	0.0197 (12)	0.0192 (13)	−0.0003 (10)	−0.0054 (11)	0.0041 (10)
C3	0.0295 (14)	0.0298 (14)	0.0247 (14)	−0.0020 (11)	−0.0020 (11)	0.0056 (11)
C4	0.0358 (15)	0.0314 (15)	0.0281 (15)	−0.0124 (12)	−0.0082 (12)	0.0076 (11)
C5	0.0568 (19)	0.0167 (13)	0.0392 (18)	−0.0072 (13)	−0.0184 (15)	0.0038 (11)
C6	0.0405 (17)	0.0220 (14)	0.058 (2)	0.0100 (12)	−0.0093 (15)	−0.0040 (13)
C7	0.0281 (14)	0.0227 (13)	0.0485 (19)	0.0004 (11)	−0.0079 (13)	0.0015 (12)
C8	0.0350 (15)	0.0251 (13)	0.0254 (15)	0.0007 (11)	−0.0057 (12)	−0.0045 (10)
C9	0.0396 (16)	0.0304 (15)	0.0381 (18)	−0.0103 (12)	−0.0052 (13)	−0.0067 (12)
C10	0.075 (2)	0.0394 (17)	0.0328 (18)	−0.0116 (17)	0.0153 (16)	−0.0100 (14)
C11	0.0220 (13)	0.0230 (13)	0.0249 (14)	−0.0017 (10)	0.0009 (10)	−0.0061 (10)
C12	0.0352 (15)	0.0384 (15)	0.0267 (15)	−0.0005 (12)	−0.0072 (12)	−0.0102 (12)
C13	0.0423 (18)	0.057 (2)	0.0397 (19)	0.0019 (15)	−0.0102 (14)	−0.0255 (15)
C14	0.0392 (19)	0.0485 (19)	0.078 (3)	−0.0093 (15)	−0.0070 (18)	−0.0400 (18)
C15	0.055 (2)	0.0299 (16)	0.070 (2)	−0.0126 (15)	0.0029 (18)	−0.0121 (16)
C16	0.0400 (16)	0.0259 (14)	0.0412 (18)	−0.0112 (12)	−0.0012 (14)	−0.0032 (12)
C17	0.0255 (13)	0.0223 (12)	0.0164 (13)	−0.0034 (10)	0.0029 (10)	−0.0017 (9)
C18	0.0363 (16)	0.0433 (16)	0.0272 (16)	−0.0026 (13)	0.0015 (13)	−0.0157 (13)
C19	0.056 (2)	0.078 (3)	0.0323 (19)	−0.0080 (18)	0.0096 (16)	−0.0249 (16)
C20	0.052 (2)	0.058 (2)	0.054 (2)	0.0009 (16)	0.0267 (18)	−0.0227 (16)
C21	0.0277 (16)	0.0544 (19)	0.0431 (18)	0.0026 (14)	0.0128 (13)	−0.0054 (15)
C22	0.0239 (14)	0.0415 (16)	0.0288 (16)	0.0039 (11)	0.0042 (11)	−0.0028 (11)
C23	0.0244 (13)	0.0255 (13)	0.0191 (13)	0.0029 (10)	−0.0034 (10)	−0.0001 (10)
C24	0.0231 (14)	0.0375 (15)	0.0248 (14)	0.0003 (11)	0.0024 (11)	0.0050 (11)
C25	0.0234 (15)	0.0557 (19)	0.0392 (18)	0.0025 (13)	0.0036 (13)	−0.0005 (14)
C26	0.0328 (17)	0.065 (2)	0.0351 (18)	0.0218 (15)	−0.0037 (14)	0.0056 (15)
C27	0.0382 (17)	0.0500 (19)	0.0305 (17)	0.0164 (14)	−0.0066 (14)	0.0087 (13)
C28	0.0320 (15)	0.0349 (15)	0.0260 (15)	0.0065 (12)	−0.0016 (12)	0.0091 (11)

### Geometric parameters (Å, °)

**Table:**

Au—P1	2.2641 (6)	C14—H14B	0.9900
Au—S1	2.2998 (6)	C15—C16	1.536 (4)
S1—C1	1.755 (2)	C15—H15A	0.9900
P1—C23	1.843 (2)	C15—H15B	0.9900
P1—C17	1.844 (2)	C16—H16A	0.9900
P1—C11	1.847 (2)	C16—H16B	0.9900
O1—C1	1.361 (3)	C17—C22	1.527 (3)
O1—C8	1.458 (3)	C17—C18	1.530 (3)
N1—C1	1.263 (3)	C17—H17	1.0000
N1—C2	1.414 (3)	C18—C19	1.532 (4)
C2—C3	1.382 (3)	C18—H18A	0.9900
C2—C7	1.383 (3)	C18—H18B	0.9900
C3—C4	1.380 (3)	C19—C20	1.504 (5)
C3—H3	0.9500	C19—H19A	0.9900
C4—C5	1.379 (4)	C19—H19B	0.9900
C4—H4	0.9500	C20—C21	1.515 (4)
C5—C6	1.377 (4)	C20—H20A	0.9900
C5—H5	0.9500	C20—H20B	0.9900
C6—C7	1.384 (3)	C21—C22	1.528 (4)
C6—H6	0.9500	C21—H21A	0.9900
C7—H7	0.9500	C21—H21B	0.9900
C8—C10	1.500 (4)	C22—H22A	0.9900
C8—C9	1.508 (3)	C22—H22B	0.9900
C8—H8	1.0000	C23—C28	1.518 (3)
C9—H9A	0.9800	C23—C24	1.533 (3)
C9—H9B	0.9800	C23—H23	1.0000
C9—H9C	0.9800	C24—C25	1.522 (4)
C10—H10A	0.9800	C24—H24A	0.9900
C10—H10B	0.9800	C24—H24B	0.9900
C10—H10C	0.9800	C25—C26	1.535 (4)
C11—C12	1.527 (3)	C25—H25A	0.9900
C11—C16	1.538 (3)	C25—H25B	0.9900
C11—H11	1.0000	C26—C27	1.512 (4)
C12—C13	1.524 (3)	C26—H26A	0.9900
C12—H12A	0.9900	C26—H26B	0.9900
C12—H12B	0.9900	C27—C28	1.525 (4)
C13—C14	1.517 (5)	C27—H27A	0.9900
C13—H13A	0.9900	C27—H27B	0.9900
C13—H13B	0.9900	C28—H28A	0.9900
C14—C15	1.515 (5)	C28—H28B	0.9900
C14—H14A	0.9900		
			
P1—Au—S1	174.54 (2)	C15—C16—C11	110.0 (2)
C1—S1—Au	106.46 (8)	C15—C16—H16A	109.7
C23—P1—C17	106.88 (11)	C11—C16—H16A	109.7
C23—P1—C11	106.67 (11)	C15—C16—H16B	109.7
C17—P1—C11	108.45 (11)	C11—C16—H16B	109.7
C23—P1—Au	110.38 (8)	H16A—C16—H16B	108.2
C17—P1—Au	110.91 (8)	C22—C17—C18	110.2 (2)
C11—P1—Au	113.27 (8)	C22—C17—P1	109.94 (16)
C1—O1—C8	117.25 (17)	C18—C17—P1	111.10 (17)
C1—N1—C2	120.1 (2)	C22—C17—H17	108.5
N1—C1—O1	120.2 (2)	C18—C17—H17	108.5
N1—C1—S1	126.33 (19)	P1—C17—H17	108.5
O1—C1—S1	113.51 (16)	C17—C18—C19	110.2 (2)
C3—C2—C7	118.7 (2)	C17—C18—H18A	109.6
C3—C2—N1	122.4 (2)	C19—C18—H18A	109.6
C7—C2—N1	118.7 (2)	C17—C18—H18B	109.6
C4—C3—C2	120.7 (2)	C19—C18—H18B	109.6
C4—C3—H3	119.7	H18A—C18—H18B	108.1
C2—C3—H3	119.7	C20—C19—C18	112.5 (3)
C5—C4—C3	120.5 (3)	C20—C19—H19A	109.1
C5—C4—H4	119.7	C18—C19—H19A	109.1
C3—C4—H4	119.7	C20—C19—H19B	109.1
C6—C5—C4	119.0 (2)	C18—C19—H19B	109.1
C6—C5—H5	120.5	H19A—C19—H19B	107.8
C4—C5—H5	120.5	C19—C20—C21	110.8 (3)
C5—C6—C7	120.6 (3)	C19—C20—H20A	109.5
C5—C6—H6	119.7	C21—C20—H20A	109.5
C7—C6—H6	119.7	C19—C20—H20B	109.5
C2—C7—C6	120.5 (2)	C21—C20—H20B	109.5
C2—C7—H7	119.8	H20A—C20—H20B	108.1
C6—C7—H7	119.8	C20—C21—C22	110.6 (2)
O1—C8—C10	110.2 (2)	C20—C21—H21A	109.5
O1—C8—C9	104.6 (2)	C22—C21—H21A	109.5
C10—C8—C9	112.9 (2)	C20—C21—H21B	109.5
O1—C8—H8	109.7	C22—C21—H21B	109.5
C10—C8—H8	109.7	H21A—C21—H21B	108.1
C9—C8—H8	109.7	C17—C22—C21	112.0 (2)
C8—C9—H9A	109.5	C17—C22—H22A	109.2
C8—C9—H9B	109.5	C21—C22—H22A	109.2
H9A—C9—H9B	109.5	C17—C22—H22B	109.2
C8—C9—H9C	109.5	C21—C22—H22B	109.2
H9A—C9—H9C	109.5	H22A—C22—H22B	107.9
H9B—C9—H9C	109.5	C28—C23—C24	111.2 (2)
C8—C10—H10A	109.5	C28—C23—P1	115.66 (18)
C8—C10—H10B	109.5	C24—C23—P1	110.57 (17)
H10A—C10—H10B	109.5	C28—C23—H23	106.3
C8—C10—H10C	109.5	C24—C23—H23	106.3
H10A—C10—H10C	109.5	P1—C23—H23	106.3
H10B—C10—H10C	109.5	C25—C24—C23	110.9 (2)
C12—C11—C16	110.5 (2)	C25—C24—H24A	109.5
C12—C11—P1	112.33 (17)	C23—C24—H24A	109.5
C16—C11—P1	115.17 (18)	C25—C24—H24B	109.5
C12—C11—H11	106.0	C23—C24—H24B	109.5
C16—C11—H11	106.0	H24A—C24—H24B	108.1
P1—C11—H11	106.0	C24—C25—C26	111.0 (2)
C13—C12—C11	110.8 (2)	C24—C25—H25A	109.4
C13—C12—H12A	109.5	C26—C25—H25A	109.4
C11—C12—H12A	109.5	C24—C25—H25B	109.4
C13—C12—H12B	109.5	C26—C25—H25B	109.4
C11—C12—H12B	109.5	H25A—C25—H25B	108.0
H12A—C12—H12B	108.1	C27—C26—C25	111.1 (2)
C14—C13—C12	111.6 (2)	C27—C26—H26A	109.4
C14—C13—H13A	109.3	C25—C26—H26A	109.4
C12—C13—H13A	109.3	C27—C26—H26B	109.4
C14—C13—H13B	109.3	C25—C26—H26B	109.4
C12—C13—H13B	109.3	H26A—C26—H26B	108.0
H13A—C13—H13B	108.0	C26—C27—C28	110.9 (2)
C15—C14—C13	111.1 (2)	C26—C27—H27A	109.5
C15—C14—H14A	109.4	C28—C27—H27A	109.5
C13—C14—H14A	109.4	C26—C27—H27B	109.5
C15—C14—H14B	109.4	C28—C27—H27B	109.5
C13—C14—H14B	109.4	H27A—C27—H27B	108.1
H14A—C14—H14B	108.0	C23—C28—C27	111.2 (2)
C14—C15—C16	111.4 (3)	C23—C28—H28A	109.4
C14—C15—H15A	109.4	C27—C28—H28A	109.4
C16—C15—H15A	109.4	C23—C28—H28B	109.4
C14—C15—H15B	109.4	C27—C28—H28B	109.4
C16—C15—H15B	109.4	H28A—C28—H28B	108.0
H15A—C15—H15B	108.0		
			
P1—Au—S1—C1	−165.2 (2)	C14—C15—C16—C11	−56.7 (3)
S1—Au—P1—C23	−58.3 (3)	C12—C11—C16—C15	56.9 (3)
S1—Au—P1—C17	59.9 (3)	P1—C11—C16—C15	−174.5 (2)
C2—N1—C1—O1	−177.9 (2)	C23—P1—C17—C22	174.41 (17)
C2—N1—C1—S1	1.6 (4)	C11—P1—C17—C22	−70.93 (19)
C8—O1—C1—N1	6.6 (3)	Au—P1—C17—C22	54.05 (18)
C8—O1—C1—S1	−173.04 (17)	C23—P1—C17—C18	52.1 (2)
Au—S1—C1—N1	157.6 (2)	C11—P1—C17—C18	166.76 (18)
Au—S1—C1—O1	−22.86 (18)	Au—P1—C17—C18	−68.26 (19)
C1—N1—C2—C3	64.9 (3)	C22—C17—C18—C19	54.9 (3)
C1—N1—C2—C7	−120.5 (3)	P1—C17—C18—C19	177.0 (2)
C7—C2—C3—C4	1.8 (4)	C17—C18—C19—C20	−56.2 (4)
N1—C2—C3—C4	176.4 (2)	C18—C19—C20—C21	56.6 (4)
C2—C3—C4—C5	0.0 (4)	C19—C20—C21—C22	−55.6 (4)
C3—C4—C5—C6	−1.2 (4)	C18—C17—C22—C21	−56.1 (3)
C4—C5—C6—C7	0.7 (4)	P1—C17—C22—C21	−178.89 (18)
C3—C2—C7—C6	−2.3 (4)	C20—C21—C22—C17	56.3 (3)
N1—C2—C7—C6	−177.1 (2)	C17—P1—C23—C28	61.4 (2)
C5—C6—C7—C2	1.0 (4)	C11—P1—C23—C28	−54.4 (2)
C1—O1—C8—C10	73.4 (3)	Au—P1—C23—C28	−177.88 (16)
C1—O1—C8—C9	−165.0 (2)	C17—P1—C23—C24	−171.08 (17)
C23—P1—C11—C12	−146.24 (18)	C11—P1—C23—C24	73.06 (19)
C17—P1—C11—C12	99.0 (2)	Au—P1—C23—C24	−50.38 (18)
Au—P1—C11—C12	−24.6 (2)	C28—C23—C24—C25	−55.4 (3)
C23—P1—C11—C16	86.1 (2)	P1—C23—C24—C25	174.72 (18)
C17—P1—C11—C16	−28.7 (2)	C23—C24—C25—C26	55.2 (3)
Au—P1—C11—C16	−152.29 (16)	C24—C25—C26—C27	−56.2 (3)
C16—C11—C12—C13	−56.7 (3)	C25—C26—C27—C28	56.4 (3)
P1—C11—C12—C13	173.13 (19)	C24—C23—C28—C27	55.9 (3)
C11—C12—C13—C14	56.0 (3)	P1—C23—C28—C27	−176.94 (19)
C12—C13—C14—C15	−55.4 (3)	C26—C27—C28—C23	−56.6 (3)
C13—C14—C15—C16	56.0 (4)		

### Hydrogen-bond geometry (Å, °)

**Table:**

*D*—H···*A*	*D*—H	H···*A*	*D*···*A*	*D*—H···*A*
C4—H4···O1^i^	0.95	2.48	3.367 (3)	156

Symmetry codes: (i) −*x*+1/2, *y*+1/2, *z*.
